# Unlock digital health promotion in LMICs to benefit the youth

**DOI:** 10.1371/journal.pdig.0000315

**Published:** 2023-08-04

**Authors:** Agata Ferretti, Effy Vayena, Alessandro Blasimme

**Affiliations:** Department of Health Sciences and Technology, ETH Zurich, Zurich, Switzerland; University of Bayreuth: Universitat Bayreuth, GERMANY

## Abstract

As digital technologies such as smartphones and fitness bands become more ubiquitous, individuals can engage in self-monitoring and self-care, gaining greater control over their health trajectories along the life-course. These technologies appeal particularly to young people, who are more familiar with digital devices. How this digital transformation facilitates health promotion is therefore a topic of animated debate. However, most research to date focuses on the promise and peril of digital health promotion (DHP) in high-income settings, while DHP in low- and middle-income countries (LMICs) remain largely unexplored. This narrative review aims to fill this gap by critically examining key ethical challenges of implementing DHP in LMICs, with a focus on young people. In the existing literature, we identified potential impediments as well as enabling conditions. Aspects to consider in unlocking the potential of DHP include (1) addressing the digital divide and structural injustice in data-related practices; (2) engaging the target population and responding to their specific needs given their economic, cultural, and social contexts; (3) monitoring the quality and impact of DHP over time; and (4) improving responsible technology governance and its implementation. Addressing these concerns could result in meaningful health benefits for populations lacking access to more conventional healthcare resources.

## Author summary

We conducted a narrative review exploring the ethical challenges of using digital technologies to promote health for young people living in low- and middle-income countries (LMICs). While smartphones and fitness bands are increasingly common, our understanding of their potential to improve health outcomes in LMICs, especially for young people, is still limited. Our review identifies several important factors for successful digital health promotion (DHP) in LMICs. First, we must address unfair data practices and the gap in access to technology. Second, it is crucial to engage young people and consider their specific needs within their economic, cultural, and social contexts. Third, monitoring the quality and impact of DHP over time is important to ensure its effectiveness. Finally, responsible technology governance and implementation needs improvement. Addressing these concerns could lead to significant health benefits for populations with limited access to traditional healthcare resources. This review contributes to the ongoing discussion on the benefits of DHP for youth, highlighting the importance of further research and action in LMICs.

## 1. Introduction

The concept of health promotion, defined as the “process of enabling people to increase control over their health and its determinants, and thereby improve their health” [1], emerged in the early 1970s in Canada, following the realization on the part of public health authorities that despite significant increases in healthcare costs and investments, low health quality persisted among a portion of the population. The health promotion movement, which has since grown rapidly internationally, highlighted the inadequacy of existing healthcare models [2], calling for more patient engagement and a wider holistic view of health, one that considers all spheres of life and takes a multisectoral approach [3]. These ideas form the foundation of the seminal WHO Ottawa Charter for Health Promotion (1986), stating that “health promotion focuses on achieving equity in health.”

Health promotion continues to play a key role in global health policy. Although new individual and public health challenges have emerged since the adoption of the Ottawa Charter for Health Promotion in 1986, health promotion’s core message has remained relevant [4]. That is, health should be a means to a better life, rather than the goal of life itself [5].

Assuming appropriate and fairly distributed conditions that make health-promoting choices possible, individuals play a key role in sustaining their own health. In this regard, it is interesting to note that the Ottawa Charter definition, by putting the spotlight on personal agency, quickly gave rise to the idea of self-care as an integral aspect of health promotion [6]. Indeed, all those activities directed at strengthening individual health literacy and awareness, as well as individuals’ activities to manage and maintain their health or address and prevent the root causes of illness, may promote health. Health promotion acknowledges both the importance of conventional disease prevention strategies and the social, economic, and environmental determinants over which individuals have limited control [7]. While highlighting the role of social determinants of health, health promotion also emphasizes the value of individual choices and behaviors such as embracing an active lifestyle, avoiding drugs and tobacco, maintaining a healthy diet, managing stress, and coping with mental health issues.

The literature reports how the success of health promotion programs is often the result of a “bottom-up” approach that places healthy individuals and communities at the center, as responsible for and actively in charge of their own health, as opposed to passive recipients of decisions by health authorities [8]. This shift towards actively empowering people to stay fit and in good health presents an opportunity to overcome potential limitations of health systems to provide adequate services, especially in resource-poor settings. The concept of self-care offers opportunities for better health even in contexts where primary care is difficult to access, medical personnel are scarce, or management of acute illness is cost prohibitive; hence, WHO’s endorsement of the value of self-care practices for alleviating global health burdens and achieving the Sustainable Development Goals (SDGs) [9,10].

Building on this background, this narrative review aims to illustrate how health promotion practices have been influenced and shaped by the emergence of new technologies. The scope of this piece is first to provide a broad perspective on opportunities presented by digital health promotion (DHP), and then to focus on the relevance of DHP for youth in low- and middle-income settings. Finally, we highlight enabling factors necessary to realize the potential of DHP for youth in developing economies.

## 2. The rise of digital health promotion—DHP

Since the early 2000s, together with the digitization of the health sector and the explosion of the use of big data for health [11], the increasing availability of digital technologies and their integration into self-care programs have reshaped the concept of health promotion, giving rise to DHP [12]. Although a clear definition of DHP is still lacking, for the purposes of this review, we define DHP as digital technologies utilized to propagate health awareness, or digital strategies and campaigns which engage and enable users to monitor their own lifestyle and health habits, fostering healthy behavior change. These technologies include social media platforms, webpages, mobile and web apps, wearable devices, computer/mobile games, AI powered chatbots, and texting services. Recent WHO initiatives promoting the worldwide adoption of digital health underscore the unprecedented potential of such technologies for improving self-care and public benefit [13,14]. Indeed, the growing success of DHP is due to several factors.

### 2.1. Expanding access to health promotion services

The widespread availability of the internet and smartphones enables individuals to search for and engage with health information everywhere. Information is available 24/7, privately, and without involving a healthcare professional. In settings with a shortage of healthcare workers or public health services, DHP can be especially useful for non-urgent health matters. The flexible and discreet nature of these tools makes them compatible with a range of time and lifestyle demands [15]. Digital platforms allow individuals to discuss health-sensitive topics, share their experiences, and seek support in discreet or anonymous ways [16]. For example, during the COVID-19 pandemic, web-based platforms emerged to support mental health. These programs aimed to reinforce positive habits by offering personalized emotional coaching that preserved user privacy and feedback via chatbots, peer support platforms, and content gamification [17,18].

### 2.2. Offering personalized services

DHP strategies are characterized by their user centricity [19]. Users generate data in 2 ways: through active input (e.g., recording body weight as measured by a smart scale, calculating calorie intake by self-reporting food consumption into an app) or passive input, via tracking devices [20,21]. These tools (such as a smartphone in the pocket, a smartwatch, or movement sensor under the mattress) run on a constant basis, creating measurements of temperature, breathing, heart rate, physical activity, blood pressure, body composition, and BMI [22]. Health apps make this health information accessible to individuals, aiming to encouraging physical activity or other positive health habits [23,24]. Yet, a primary appeal of DHP technologies lies in their ability to provide users with tailored feedback (in the form of messages, reminders, or resources) addressing individual health goals [25]. By leveraging individual preferences and adjusting feedback accordingly in real time, DHP holds potential for greater impact than traditional “one size fits all” public health promotion strategies.

### 2.3. Supporting healthy lifestyles

DHP applications aim to increase awareness of health habits, and to support users in health-related decisions, aiming to prevent future decline in health status. Studies have described how digital health technologies engage individuals, resulting in active behavior change [26,27]. Through gamification and creation of avatars, for example, DHP applications seek to nudge consumers to embrace “healthier lifestyles,” while increasing their information receptivity and retention rates [28,29]. Leveraging reward strategies and social elements, DHP not only engages users, but also keeps them motivated to achieve their health goals [30,31].

### 2.4. Containing costs

DHP interventions are an attractive choice for addressing health risks in part because the scaling costs are low. Because DHP strategies can be implemented via consumer technologies that most people already own, it is increasingly feasible to introduce initiatives targeting entire communities [32]. Examples include DHP strategies to improve health literacy, contact tracing apps alerting users of locations with high COVID-19 infection rates, or apps promoting immunization. Apart from the initial cost of creating software or designing the technology, upscaling a digital intervention is relatively inexpensive for developers [33]. By avoiding the significant expense often associated with traditional health promotion strategies, DHP have potential to reach a broader audience, maximizing impact. DHP is increasingly affordable, even for vulnerable individuals who face financial, cultural, logistical, or information barriers to accessing traditional healthcare or health promotion services [12].

## 3. DHP for adolescents and young adults (AYAs)

Adolescents and young adults (AYAs)—namely people 10 to 24 years old—account for more than 1.8 billion people globally, or a quarter of the world’s population [34]. A recent review of the literature revealed that in high-income countries (HICs), AYAs use digital technologies extensively to monitor their health and promote physical and mental well-being [35]. This finding aligns with earlier research reporting the feasibility, efficacy, and cost-effectiveness of digital interventions to promote health among young people in advanced economies [36,37]. In light of such trends, over the last years important international initiatives (such as The Lancet Commission on Adolescent Health and Wellbeing) have focused on bringing young people into the discussion about digital initiatives, with the aim of achieving the SDGs [38,39].

For the field of health promotion specifically, young people may represent a breaking point with the past. Many diseases and behaviors that develop during adolescence and young adulthood carry negative consequences throughout the lifespan [40]. For example, malnutrition and lack of adequate activity are connected to the exponential increase in youth obesity in Kenya and Ghana, with serious consequences for future health (cardiovascular diseases and diabetes) [41,42]. Similarly, early pregnancies and sexually transmitted diseases (such as HIV), unaddressed mental health issues (such as trauma and depression), use of tobacco and substances (i.e., alcohol and drugs), violence, and poor physical activity constitute the greatest health risks faced by AYAs worldwide [40,43]. Targeting DHP solutions to a still healthy population may improve young peoples’ behaviors and thus their state of health, both immediately and in the long term [44].

The available academic literature reports several reasons why DHP finds fertile ground among young people. First, youth appreciate the breadth of information available via digital media and the fact that this information is available at any time and without involving intermediaries (e.g., physicians) [45]. Internet connectivity enables access to health-related information and services even in contexts with geographical barriers or health professionals’ shortage [46]. Secondly, content is often personalized and engaging (for example, through gamification). Thus, the target audience is motivated to engage with the information, encouraging receptiveness to the subject at hand [47]. In addition, young people appreciate the ability to connect with peers, creating support groups while maintaining anonymity, and thus preventing potential stigmatization [48]. Finally, the low cost of such technologies allows the younger generation to access health promotion services without incurring out-of-pocket expenses [12].

## 4. Shedding light on DHP in LMICs

While the contribution of digital technologies to health promotion has been critically examined in the context of HICs, research on the potential and challenges for implementing DHP in low- and middle-income countries (LMICs) is limited [49,50]. One possible explanation is that digital health is often associated with sophisticated forms of healthcare and technologies (e.g., expensive wearable devices powered by artificial intelligence or machine learning-driven interpretation of medical images), which may be less accessible in LMIC settings. Yet, health promotion can also occur via other more common forms of digital media, from text messages to social media platforms [20,51]. So far, however, only a few studies have reported on the potential of these DHPs in emerging economies. Among them, for example, research conducted in South Africa shows how social media can rapidly disseminate health behavior information throughout the population [52]. Similarly, researchers in Tanzania have tested the effectiveness of animated health videos and interactive platforms for public health promotion in relation to HIV and tuberculosis [53].

In the coming years, young people in LMICs will have increasing access to technologies, and DHP adoption will no longer be weighted towards wealthy countries [54]. Already, data show a general increase in the uptake of smartphones in LMICs. Internet coverage continues to grow in sub-Saharan Africa, with a substantial increase during the COVID-19 pandemic [55]. Internet access has increased for youth in particular: in 2020, 40% of youth (15 to 24 years old) in Africa accessed the internet, compared with 27% of the general population [56]. Moreover, as digital natives, young people will be more tech-savvy and comfortable in adopting DHPs than older generations [57].

Young people across borders seem eager to harness the benefits of technology and make their voices heard concerning their needs [58,59]. Today’s youth demonstrate awareness not only of their role in maintaining their own health, but also in leveraging technology to address pressing public health challenges [60]. Examples of such initiatives include the Young Experts Tech for Health and the Lancet Youth Network. These youth networks are driving action toward meaningful youth engagement in technology design, affordable training in digital skills, and broader access to health education and services. The Ndola Youth Resource Centre, a youth-led organization based in Zambia, co-developed the initial content for TuneMe, an online health promotion platform tailored to the health needs of AYAs of 7 African countries [61]. Its features include a collection of resources on HIV, STIs, reproductive systems, and safe sexual behaviors, as well as a direct booking system for screening services, and a platform to anonymously discuss issues of sexuality.

Despite the anticipated benefits, significant knowledge gaps exist about how digital technology can effectively foster young people’s sexual and reproductive health, mental health, or healthy lifestyle behaviors in LMICs [62,63]. Particularly, rigorous assessment of the perspectives, insights, and concerns of youth about DHP are still largely missing from the current dialogue. Since major benefits for individual and global health are at stake, DHP strategies for youth deserve more careful exploration.

## 5. Main challenges and risks

DHP initiatives have gained momentum among public health agencies, NGOs, and international organizations due to their potential for realizing and expanding access to health promotion also in LMICs (and particularly among youth). For example, the “Youth-centred digital health interventions” document published by WHO in 2020 aimed to foster and guide the development and implementation of digital health solutions for young people worldwide, reporting on successful case studies from Africa to South America [64]. Alongside the promises, the literature on health promotion, together with that on digital health, offers insights into the dangers and ethical challenges of these technologies [65]. Such concerns must be investigated and addressed promptly to avoid introducing technologies that bring more harm than good.

### 5.1. Access and allocation of technologies

The digital divide—understood as the discrepancy in access to digital infrastructure and literacy across settings and population groups—poses limits to the opportunities offered by DHP. While this is a well-known problem in the field of digital health, it is even more pronounced in contexts where economic resources are limited, and inequalities are significant [66]. In LMICs, the digital divide problem is stratified.

First, some areas within resource-poor countries may lack reliable and interoperable information infrastructure and appropriate technologies (e.g., broadband and stable internet) due to economic, geographic, political, and cultural barriers. Internet infrastructure is uneven across and within countries; for example, between rural and urban areas [67]. Such technical limitations can result in tools that cannot be utilized, such as apps that require constant network access for data synchronization.

Second, when infrastructures and technologies do exist, they are often not equally accessible across the population. As socioeconomic and cultural barriers stand between the availability of health-promoting technologies and their effective use, the most vulnerable demographic groups face the greatest disadvantage (e.g., women, the elderly, ethnic minorities, the poorest, and the least literate) [68]. Because health promotion content is increasingly delivered digitally, population segments that lack digital literacy and have little experience with digital technologies may be excluded [69]. Despite the growing availability of infrastructure and tech exposure, lack of digital literacy still represents the main barrier to youth benefitting from digital health innovation [70]. Additionally, although much of the content offered by DHP is in English, vulnerable populations—including AYAs in LMICs—predominantly speak local languages [71]. Hence, the potential inability to benefit from such services and understand health messages [72]. Even more so, solutions that require a higher level of reading skills but lack gamification and interactive design aspects, as well as relevant cultural cues, would not be adopted by those with limited (digital) literacy [73]. Inability to afford expensive data plans is a further reason why AYAs might not adopt DHP. Accessing DHP in public spaces with free Wi-Fi can be a solution, but it may also create potential discomfort due to the sensitive nature of the topic; lack of access from home or other safe spaces might result in young people not taking advantage of the technology at all [74,75].

Third, contrary to what is generally the case in HICs, digital devices such as mobile phones are not necessarily available to single users in LMICs [76]. Oftentimes, multiple members of the same family share the same device, to limit costs of the hardware and the data plan. In order to maximize health outcomes and to minimize privacy-related risks, it is thus important to design DHP applications in a way that takes into account how digital platforms are actually used in a specific context.

Finally, the lack of equal access to digital technologies leads to data poverty, which results in incomplete and nonrepresentative health datasets [77]. These biased datasets, in turn, may be used in the assessment and evaluation of DHP technologies, potentially creating inefficient, or even unsafe, implementation. That DHP may have poor outcomes is even more worrisome from a public health perspective, with potential ramifications of harmful health consequences and an increase in inequality, to the disadvantage of already underprivileged groups.

### 5.2. Fair distribution of benefits

Amid the discussion over the use of digital health to improve global health and expand health promotion strategies, some authors have raised concerns about the risk of digital and data colonialism as an effect of structural injustice [78]. A growing number of private companies from HICs are establishing themselves in the Global South by offering products, technological infrastructure, and human expertise in digital health. As a result, technologies and computational methods created in developed economies are deployed to acquire data from populations in developing economies [79,80]. Just as historical colonial practices involved the appropriation of land, the new form of data colonialism undermines individuals’ sovereignty over their data and communities’ capacity to address context-specific health needs [81].

While digital tools and large technology companies can potentially complement the role of public health institutions, the risk of misusing the health data of local populations generated through these new technologies is increased. In the name of open data access and to address the data poverty issue, companies transfer data acquired via digital technologies in the Global South to the West for research. While this harvested data can generate health-related knowledge, it primarily serves to create profit for those in control of the data, as corporations may sell the extracted information to third parties for consumer and business services [82,83].

Such data and techno colonialist practices are not unique to the private sector, but occur in academic health research as well. For example, in the field of global health, northern research institutions have for decades collected and monitored malaria data from endemic areas of Africa, without involving indigenous people in data analysis or presentation of results [84]. Today, DHP tools enable collect vast amounts of data about their users, but at the same time such data may be interpreted in developed countries by researchers lacking adequate contextualization. Therefore, while institutions that draw on data knowledge can respond to people’s health needs in a timely manner through specific health promotion solutions, they also contribute to structural inequalities by maintaining control over data and exacerbating exploitation mechanisms [79]. The asymmetry of power described in this section between those who collect the data and those who provide it raises a burning yet unanswered question: Whose interests are served or denied when applying DHP strategies in LMICs?

### 5.3. Privacy and misinformation

DHP technologies may require or enable gathering and analyzing significant amounts of data. Some health data governance frameworks encourage the use of personal data as a means of promoting and improving health [85,86]. However, DH technologies can also put users’ privacy and safety at risk. Because in most countries, these direct-to-consumer technologies are not vetted by a health or data authority, there is a risk for irresponsible data use. For example, fitness and wellness apps may share sensitive data with third parties without the user’s permission or knowledge, and collect data points beyond those strictly necessary for their function. In addition, the personal nature of health information raises additional concerns about data leakage, disclosures, or misuse. Data could be used by marketing companies for targeted advertisements, by cybercriminals for blackmail and profit, or by governments to identify and prosecute certain minorities. This is especially relevant in contexts where data governance is not established or does not extend to all data uses; data security mechanisms are shaky, and individual and collective stakes are high. As of 2022, only 48% of the least developed countries have data protection and privacy legislation in place [87].

Breaches of health data collected via DHP can expose young people in particular to the risks of cyberbullying and online harassment. Because AYAs are the most active online and potentially least aware about how to protect themselves online, they are exceptionally vulnerable to such threats. For example, in Finland in 2020, sensitive data from students who adopted an easy-to-use app to boost mental health were leaked by hackers whose ransom requests were denied. Students experienced emotional, social, and reputational damage as a result, to the extent that some users changed phone numbers or social security numbers, or removed their presence from the web [88].

Because most DHPs are not considered medical devices, they do not have to abide by traditional oversight mechanisms. Thus, no regulatory authority is responsible to ensure that DHP technologies offer accurate evidence-based information, or recommendations that are demonstrably safe and effective. Misinformation and false sources could in particular harm young people, who are not in the habit of fact-checking online content (e.g., YouTube videos, Instagram photos, or app recommendations). Similarly, misleading messages embedded in DHP may lead especially those with low levels of health literacy to engage in behaviors that may turn out as useless or even harmful. For example, AYAs might be persuaded by catchy visuals to smoke e-cigarettes instead of traditional cigarettes, if the former is labeled on social media as a healthier and fitness-enhancing alternative. However, scientifically speaking, this information would be misleading, as both types of cigarettes contain nicotine and can harm health. Therefore, questions persist about who is responsible for the negative consequences of unsafe and unreliable technologies, in the absence of responsible innovation regulations.

### 5.4. The empowerment paradox

Some authors have challenged the value of digital health technologies, with an argument rooted in a sociological critique of the neoliberal approach to healthcare [20,89]. According to this view, a main limitation of DHP is its focus on individual responsibility, with health promotion framed as an individual effort, and a lack of attention to socioeconomic determinants of health. This critique asserts that health promotion should not focus on changing individual behaviors, but instead address the power dynamics that indirectly influence the health behaviors of individuals and communities.

Accordingly, some scholars argue that DHP is more aptly described as the digital re-creation of traditional health communication strategies [90]. While technology has been incorporated into traditional public health campaigns for some time, the actual implementation of health behavior is left to individuals. Health authorities might promote the use of apps and mobile services to keep physical fitness and mental health, but these tools are not broadly integrated into healthcare systems, and associated costs are borne by individuals [91]. Hence, DHP might not be the vehicle for addressing systemic issues and prioritizing the needs of underserved population segments. On the contrary, according to this critique, DHP (and digital health more generally) could exacerbate inequalities, discriminating vulnerable groups.

This sociological critique also disputes the concept of empowerment at the core of DHP. Behind the promise of more independence and self-determination, so the argument goes, lies the risk for surveillance and control. Individuals are not only nudged, but also rather manipulated towards a specific health goal, significantly limiting individual autonomy. Some authors in particular have argued that these technological approaches to health promotion translate, in practice, to a patronizing attitude of companies and government institutions towards citizens. Institutions impose health goals and priorities as defined by a system of surveillance, data analysis, rules defined by developers, and market laws, as opposed to the needs and preferences of individuals and communities. DHP technologies, therefore, far from serving autonomy, offer users only the illusion of individual control over their health trajectory.

## 6. Considerations to unlock the potential of DHP for AYAs in LMICs

While underestimating the complexities of DHP implementation could worsen conditions for the most marginalized and worsen health disparities, failing to recognize the possibilities offered by new technologies will leave the health needs of many unmet.

### 6.1. Striving for empowering DHP

Health promotion’s overarching aspiration is to address sociopolitical determinants of health; this aim can be facilitated by the adoption of new technologies that promote self-care and well-being. From an ethical standpoint, helping people to become more aware of their health, and to take appropriate action based on that knowledge, is justified as an attempt to promote universally valued aims such as being in good health and avoid preventable health risks. Thanks to DHP, people may improve their life and health, making changes step by step. Individuals can boost their knowledge of health and wellness, learn about scientifically verified activities for staying healthy, and be supported in their efforts to reduce health risks. Especially in resource-poor settings, DHP can mean a lot for people who lack access to other reliable sources of health information or to healthcare services altogether. Still, we argue that individual responsibility for health is not a substitute for governments to provide the necessary resources and conditions that allow people to maintain healthy lives.

Therefore, we resist a divisive interpretation of DHP that overstates either the importance of the top-down approach (focused on the role of governments and institutions to address complex issues) or the bottom-up approach (focused on the individual responsibility to take actions on a daily basis). Instead, we respond with a cautious but proactive attitude to the adoption of digital technologies for health promotion.

This perspective is aimed at synergistically holding two different visions of health promotion, which are not mutually exclusive, but complementary. Especially in contexts where health systems suffer from systemic weaknesses and governments have limited resources at their disposal, solving complex problems takes time. Therefore, while working to address the social determinants of health, targeted low-cost DHP interventions could provide a timely response to the needs of a specific population. Although these interventions may only be accessible to certain groups (for example, the youth in a community), they could still progressively bring equity and positive outcomes for the whole population. By supporting some individuals, DHP can lessen the burden on the healthcare system, while allowing for more equitable redistribution of public resources. Namely, public health institutions could allocate more resources towards meeting the needs of those who, either by choice or out of necessity, do not use DHP technologies.

**Fig 1:**
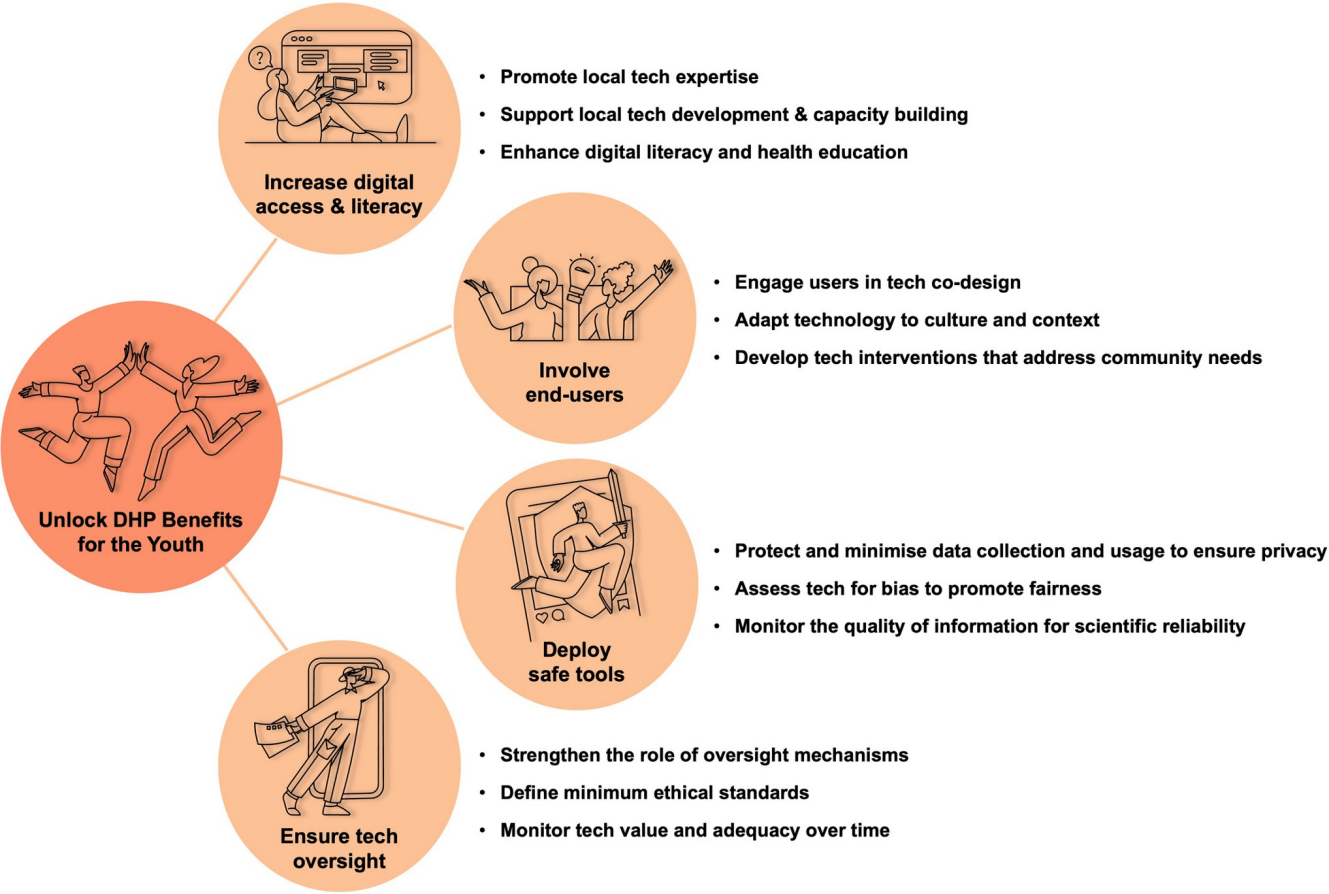
Conditions reported in the literature to facilitate the success of DHP in LMICs.

That said, the literature reports several conditions that health institutions and DHP developers should meet to facilitate the success of DHP in LMICs and ensure that the benefits of technology are equitably distributed among stakeholders (see Fig 1).

### 6.2. Increase digital access and literacy

Focusing on the digital divide, international institutions have called for mobile health tools (e.g., smartphones and wearables) to become more available and accessible worldwide [13]. Due to globalization and ongoing digital transformation, LMICs are gradually gaining stable access to broadband internet, modern IT infrastructure, and technologically advanced devices [92].

Yet, the private sector, nongovernmental agencies, and national governments should increase funding to lower the costs associated with newer infrastructure and services, encourage technological development, and train digital experts in the Global South. Local technology professionals and developers understand the needs of end-users, and they are best equipped to tailor solutions to those needs. For example, in LMICs it could be strategic to create health content in local languages with references to the relevant culture, as it will have the benefit of reducing the language barrier for young people, some of whom have not attended school and cannot read or write in English or another colonial foreign languages [93,94]. This would provide greater autonomy and independence from the Global North, not only regarding collection and management of data, but also for development and utilization of digital solutions [95,96].

As digital inclusion affects many aspects of living, scholars have recently argued for the addition of digital technologies (i.e., technology access and literacy) among the social determinants of health [97,98]. Despite expanding capacity building and tech access, challenges such as inadequate digital literacy may persist in low resource settings [96]. Thus, enhancing young people’s digital literacy and scientific fact checking skills, across genders and socioeconomic conditions, is a priority for reaching the full potential of DHP in LMICs.

### 6.3. Involve end-users

Only by effectively participating in the co-design of DHP interventions and associated technologies, and helping to define its purpose and rules, will young people have the opportunity to express their needs and priorities [60]. It is therefore necessary to involve prospective young users at various stages of technology development. Most importantly, individuals should be able to define their needs and consider whether and which technologies are the most appropriate to promote their health. This process must factor in cultural and social circumstances as well as local values. Enabling DHP co-creation might increase users’ trust in technology and willingness to use it. Developers should therefore place technologies in context and respond to users’ needs. This approach to DHP development contrasts with paternalistic and simplistic top-down methods that ignore technological relevance and reliability in context.

### 6.4. Deploy safe tools

Despite the potential for DHP to exert a transformative impact on youth health, such technological development remains a double-edged sword. Therefore, to realize its benefits, first and foremost DHP technologies must be safe [58]. Safety may be articulated in 3 ways.

First, ensuring data privacy. This entails not only minimizing data collection to the data needed to develop and deploy DHPs, but also protecting data from cyber and ransomware attacks, to safeguard individuals and groups with sensitive health characteristics (e.g., youth HIV–positive or adolescents with mental health conditions). Second, preventing the presence of unwanted bias and unfair interventions. Stakeholders must rigorously and repeatedly monitor DHPs to ensure the adequate representation of diverse demographic groups including ethnicity, gender, and wealth. Third, ensuring the scientific reliability of an intervention. While DHPs simplify complex health information, making it accessible to young people, they must not forego the nuances of scientific discourse, or worse, allow the proliferation of misinformation. Thus, alongside monitoring the quality of information provided, health institutions and DHP developers should contain any factors potentially distorting information (such as political and commercial influences) [99].

### 6.5. Ensure technology oversight

The use of DHP, particularly among vulnerable youth in developing economies, requires data governance policies and accountability mechanisms [100]. Strengthening the role of oversight instruments could ensure a more ethically aligned development and use of technologies. While the lack of adequately transparent policies and public involvement may threaten trust in entities developing DHPs, the presence of reliable, transparent, and efficient oversight systems could increase willingness to use DHPs [101]. For this reason, it is crucial to clearly define the ethical standards and boundaries of DHPs at the international level. These shared minimum standards should then be translated into more practical strategies and applied locally, according to cultural specificities and local needs. To ensure that relevant ethical standards are incorporated in DHP interventions, involved parties (e.g., funders, institutions, companies, foundations, and governmental as well as nongovernmental organizations) should appoint ad hoc ethics boards including local experts and community representatives. Such boards would specify international ethical principles in public health and health promotion into specific technical and use-related requirements to minimize risks and maximize benefit of DHP initiatives across target populations—especially in the case of vulnerable and marginalized demographics.

## 7. Conclusions

Digital health technologies become more pervasive worldwide, creating opportunities for individuals to engage in self-care and health promotion practices, intrigued by their potential benefit.

As more and more young people engage in self-care and mobilize to harness technological power for their health, the discussion about DHP urges timely research and attention. Our analysis shows the practical strength of DHPs to benefit young people and, more generally, the population of LMICs as a whole. To fully exploit its potential, however, DHP cannot be considered as a substitute for other more structural public health efforts to keep populations healthy. Rather, DHP technologies are best conceived as one of many means to achieve universal health and adequate care of individuals and populations. To this aim, institutions, experts, researchers, and global health organizations should promptly address the limitations and pitfalls of existing DHPs by engaging end-users and targeted communities in co-creative models for technology development.
